# Time needed to achieve completeness and accuracy in bedside lung ultrasound reporting in Intensive Care Unit

**DOI:** 10.1186/1757-7241-18-44

**Published:** 2010-08-12

**Authors:** Lorenzo Tutino, Giovanni Cianchi, Francesco Barbani, Stefano Batacchi, Rita Cammelli, Adriano Peris

**Affiliations:** 1 Postgraduate School of Anaesthesia and Intensive Care, Faculty of Medicine, University of Florence, Italy; 2 Anesthesia and Intensive Care Unit of Emergency Department, Careggi Teaching Hospital, Florence, Italy

## Abstract

**Background:**

The use of lung ultrasound (LUS) in ICU is increasing but ultrasonographic patterns of lung are often difficult to quantify by different operators. The aim of this study was to evaluate the accuracy and quality of LUS reporting after the introduction of a standardized electronic recording sheet.

**Methods:**

Intensivists were trained for LUS following a teaching programme. From April 2008, an electronic sheet was designed and introduced in ICU database in order to uniform LUS examination reporting. A mark from 0 to 24 has been given for each exam by two senior intensivists not involved in the survey. The mark assigned was based on completeness of a precise reporting scheme, concerning the main finding of LUS. A cut off of 15 was considered sufficiency.

**Results:**

The study comprehended 12 months of observations and a total of 637 LUS. Initially, although some improvement in the reports completeness, still the accuracy and precision of examination reporting was below 15. The time required to reach a sufficient quality was 7 months. A linear trend in physicians progress was observed.

**Conclusions:**

The uniformity in teaching programme and examinations reporting system permits to improve the level of completeness and accuracy of LUS reporting, helping physicians in following lung pathology evolution.

## Introduction

Bedside lung ultrasound can provide accurate information on lung status in critically ill patients in Intensive Care Unit (ICU) [1,2], and the important role of defining standards in critical care ultrasonography has been recently discussed [3].

Before April 2008, in the ICU of Emergency Department (Careggi Teaching Hospital, Florence, IT), bedside Lung Ultrasound (LUS) was only performed as support of invasive device positioning (central venous catheter, chest drainage), and for quantification of pleural effusions.

After April 2008, trained intensivists started to use bedside LUS on a daily basis in order to make diagnosis, to monitor chest pathologies and to improve pulmonary patterns interpretation. The present study describes the accuracy and quality curve of the LUS reporting during its method implementation.

## Methods

The study was performed in a 10-beds ICU. The ICU was equipped with two MyLab 30 CV (ESAOTE, Genova, IT) with multifrequency Convex and Linear probes. From April 2008 to April 2009, 397 patients admitted to ICU underwent LUS. A standard procedure for LUS performance was conceived in order to guarantee its reproducibility and simple consultation, and to make a uniform ultrasonographic approach to the patients [4]. The procedure defined standards for patient's positioning during the exam, areas of the thorax to be scanned, the most appropriate way to approach the thorax in order to evaluate specific pathologies and the best ultrasonographic approach to each pattern (visualization mode, ultrasonographic signs).

Furthermore, operators were invited to print pictures of all the examinated features. All intensivists were trained for bedside LUS by an internal ICU learning programme, which consisted on one day of lectures, followed by 20 hours of hands on instructions. Physicians reported competency after 3 months of proctored practice.

Ultrasonographic patterns were introduced in the electronic report sheet in the institutional ICU database (Filemaker Pro 5.5 1984-2001 Filemaker, Inc.), following a dedicated checklist. The checklist concerned information about the following ultrasonographic patterns: pleural line, diaphragm, lung parenchyma (B-lines count, consolidation), pleural effusion and pneumothorax. A blank space was left to be filled with significant details of patient's anamnesis.

**Figure 1:**
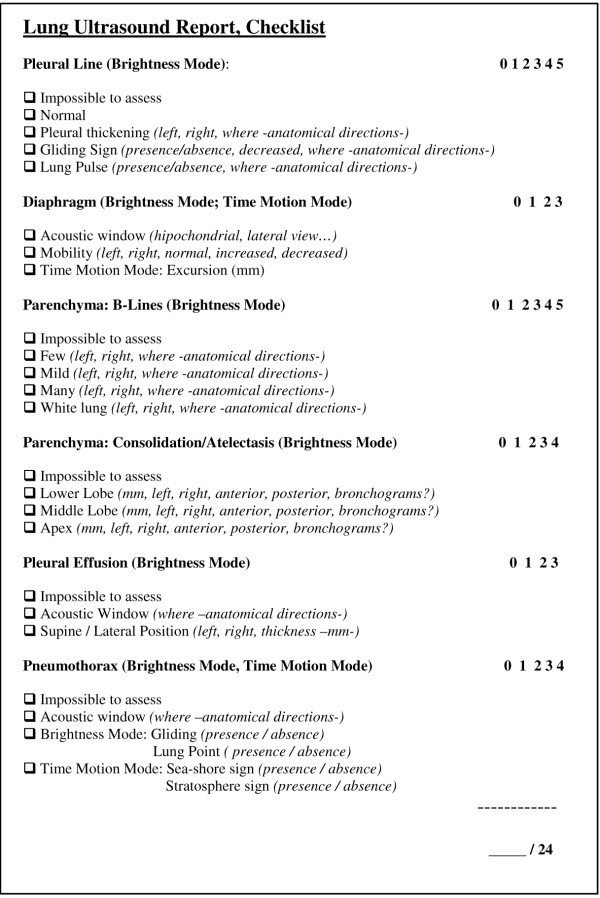
**Checklist for Lung Ultrasound reports**. Maximum mark per field was previously decided considering the number of parameters requested.

Two senior intensivists, GC and SB, checked the accuracy of the reports. They were not directly involved in the care/examination of patients included in the study. Physicians that performed the exam were not informed of the seniors' supervision. The completeness of the reports was evaluated considering the images obtained during the examination. A vote was assigned to each element of the template provided for reporting. A "0" was given for any incomplete information or any missing field. Otherwise, a "1" was assigned if the parameter was considered sufficient (Figure 1). The sum of all fields, from 0 to 24, was used to evaluate the internal ICU learning curve trend.

## Results

**Figure 2:**
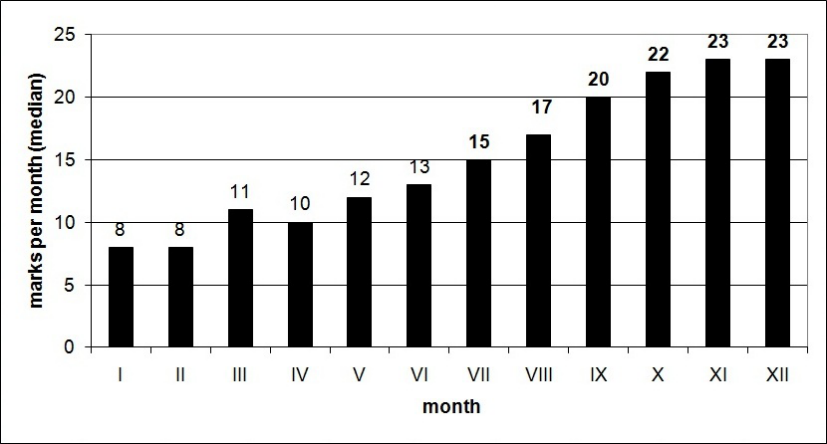
**Monthly median of marks achieved during the study period**.

During the study period (April 2008-April 2009), a total of 637 LUSs were performed, and the marks per month (median) are shown in Figure 2. Multiple LUS per patient were possible either for clinical investigation, for devices positioning, or clinical follow-up.

Significant differences regarding quality standards of LUS reporting between the first and the last month were noticed, with a constant positive trend. The worst and insufficient average vote was found in the first month, when the bedside LUS implementation had just started. To achieve sufficiency (median mark > = 15), 7 months were necessary, afterwards the standard remained high. Once data collection was completed, twelve LUS reports were randomly checked with the same method in order to confirm the marks trend, achieving a median result of 23.

The most common omissions in LUS reporting concerned three of the six considered echographic fields. The description of pleural line, B-lines and pneumothorax was generally adequate, whereas incomplete reporting was common for diaphragm motility and lung consolidations.

Diaphragm motility was often not evaluated with missing information about the quantification of the excursion.

Concerning consolidations and atelectasis, a precise definition of their extensions and anatomical localization was often lacking, compromising an adequate follow-up of the lesions.

Also bronchograms were incompletely described, therefore the diagnosis of the nature of the consolidation was often impossible. Finally, concerning pleural effusion evaluation, the statement whether it was determined in supine or lateral position, was often lacking. Nevertheless, using Balik's formula, the estimation of pleural effusion was in good relation with the effective drained volume (volume of effusion in millilitres equals the distance between lung and posterior chest wall in centimetres multiplied by 20) [5].

## Discussion

In our experience we have shown that the accuracy of LUS description improves over time by using a preset reporting module. In this descriptive study, the lack of a control group does not permit to evaluate the strength of association between electronic sheet introduction and LUS quality improvement. Moreover, in our clinical practice LUS has been widely improved over time, moving from a procedure-related tool (mere wide to pleural effusion drainage) to a wider and more frequent clinical examination method. Therefore, operators skills in LUS execution, naturally improved as they gained experience. The process of acquiring competency in ultrasound examination was already described by Schlager and co-workers in a study evaluating goal-directed ultrasound in emergency department, where that accuracy improved with gradually growing experience [6]. Kendall and Shimp demonstrated that in focused bedside ultrasound exam (abdominal right upper quadrant), the sensitivity of the exam was 100% after 25 exams performed [7]. Although gaining competency in a skill over time is a well recognized process, our study was aimed to investigate the quality of the reporting method, rather than to assess the learning curve of LUS examination. We believe that a complete LUS reporting should consider a multitude of parameters and its clinical utility correlates to accuracy of this diagnostic tool.

Considering the completeness of the reporting, with the introduction of the standardized report sheet, we report an increasing quality of the examinations during the study period, as a prompt for operators to consider all the parameters required for a complete LUS reporting.

In the same way, the standardize sheet induced operators to obtain all the required images necessary for a complete evaluation of the chest, therefore an adequate follow-up was possible comparing images taken from exams performed in sequence. Lack of proper images easily result in missing pathology or mistaking artefacts also in other fields of ultrasonography [8].

Although the scoring method we adopted is arbitrary and far from being validated, it can be regarded as a useful method to compare LUS examinations, an ever-growing exam with a strong inter-operator variability.

## Conclusions

The use of a standard report scheme for LUS can help intensivists to improve completeness and accuracy level of the examination reporting and it permits to follow the clinical course of chest pathology in ICU patients.

## Competing interests

The authors declare that they have no competing interests.

## Authors' contributions

LT wrote the manuscript, participated in the coordination of the study and took part in the internal teaching programme. GC and SB were the two seniors involved in report judgement, they also coordinated the teaching programme. FB coordinated the ICU ultrasound screening and coordinated, with the help of RC, the electronic data collection of LUS data during the study.

AP conceived the study, participated in its design and took part in the educational program. All authors read and approved the final manuscript.
